# The Characteristic Appearance of Wounds of the Intestines Made during Life

**Published:** 1882-03

**Authors:** 


### The Characteristic Appearance of Wounds of the Intestines Made During Life.

Accurate knowledge of the distinguishing marks by which to determine that a solution of continuity in an intestine is the result of violence to the living body rather than of accident during the autopsy, is of very great importance to the medico-legal inspector; and Dr. W. F. Whitney has done good service in pointing out with clearness the means of differentiation. The relation, he says, which the mucous coat bears to the edges of the wound is characteristic, and when carefully considered, will leave little doubt as to the time when the wound was inflicted. In ante-mortem wounds the edges are covered by a protrusion of this mucuous coat. The mucous membrane is loosely connected to the muscular coats, and is movable upon them to a certain extent. If all the coats of the intestine are divided, the edges of the wound will gape from the retraction of the cut muscles, and the lax mucous coat is forced through the opening by the peristaltic movement as far as its attachments will permit and curls back over the edges of the wound through the action of its elastic fibres. Once over the edges of the wound, the membrane is not retracted again, and in a few hours it develops inflammatory adhesions in its new position. Besides the new position and relations of the mucous coat, there is a slight thickening of all the coats immediately in the neighborhood of the wound from an infiltration with serum and new cells.

In a rupture or perforation from ulcer, the protrusion of the mucous membrane would not occur, because in these cases the mucous coat is extensively destroyed and fastened to the muscular coats by inflammation before the outer layers of tissues yield.—*Boston Med. and Surgical Journal.*

